# Untapped Potential for Female Patients? Comment on Lucà et al. Update on Management of Cardiovascular Diseases in Women. *J. Clin. Med.* 2022, *11*, 1176

**DOI:** 10.3390/jcm11102862

**Published:** 2022-05-19

**Authors:** Julia Kielb, Jessica Weber, Khatereh Shahjerdi, Süreyya Saffak, Leonard Baensch, Silvana Glugla, Amin Polzin, Tobias Zeus, Malte Kelm, Lisa Dannenberg

**Affiliations:** 1 Department of Cardiology, Pulmonology, and Vascular Medicine, Medical Faculty, Heinrich Heine University Düsseldorf, 40225 Düsseldorf, Germany; julia.kielb@uni-duesseldorf.de (J.K.); jessica.weber@uni-duesseldorf.de (J.W.); khatereh.shahjerdi@uni-duesseldorf.de (K.S.); suereyya.saffak@uni-duesseldorf.de (S.S.); leonard.baensch@uni-duesseldorf.de (L.B.); silvana.glugla@uni-duesseldorf.de (S.G.); amin.polzin@med.uni-duesseldorf.de (A.P.); tobias.zeus@uni-duesseldorf.de (T.Z.); malte.kelm@uni-duesseldorf.de (M.K.); 2 Cardiovascular Research Institute Düsseldorf (CARID), 40225 Düsseldorf, Germany

In the recently published review titled “Update on Management of Cardiovascular Diseases in Women”, Lucà et al. highlight sex-related differences in cardiovascular disease [1]. We read this review with great interest and aim to lead the attention of the reader to the following summarized aspects with felt open gaps for possible future analyses (Figure 1):

(1) What are the differences in risk factors and primary prevention for cardiovascular disease?
(2) What are the differences in treatment in case of acute myocardial infarction (AMI) and percutaneous coronary intervention (PCI)?
(3) What are the differences in follow-up care?

There is a lack of transfer for a one-fits-all concept for male and female patients. First, women were shown to have an altered risk profile for cardiovascular disease (CVD) with reduced incidence in the pre-menopausal state but increased rates afterward [2,3]. In this context, the importance of the menopause, hormone replacement therapy, and polycystic ovary syndrome is already highlighted in this review [1]. However, the relevance of endometriosis might also be reflected, as this was revealed as a relevant co-associated factor for CVD [4]. Moreover, the current literature lacks information about number of pregnancies or potential aborts. This can help us identify relevant mechanisms behind altered risk profile due to hormone status.

Next to sex-specific risk factors, women show increased rates of relevant CVD co-morbidities, such as anemia, chronic kidney disease, metabolic syndrome, or manifest diabetes and atrial fibrillation [5,6]. The importance of gender is reflected in the “2021 ESC Guidelines on cardiovascular disease prevention in clinical practice” guidelines with the presented SCORE2 risk estimator, which include female gender and age as relevant aspects [7]. However, co-morbidities also affect outcomes after AMI or PCI. Several studies indicated that women show worse follow-up after AMI or PCI. However, gender is only poorly represented infrequently used risk scores to predict outcomes after PCI or AMI. Moreover, reached endpoints are interestingly mainly driven by mortality, bleeding, and re-hospitalization, and not by ischemic events such as re-infarction [5,6]. In this context, balancing major adverse cerebral- and cardiovascular events (MACCE) versus bleeding is crucial. As women show increased bleeding rates, sex-specific adaption of dual antiplatelet therapy duration after stent replacement therapy shall be discussed. Nevertheless, gender is currently not included in the recommended PRECISE-DAPT or high-risk bleeding score by the Academic Research Consortium [8,9,10]. Additionally, cardiac function is a relevant follow-up parameter. In this context, women were, however, shown to have a higher systolic cardiac function than men despite the worsened outcome. This leads to the question if follow-up parameters shall be expanded with inclusion of diastolic dysfunction measurement and strain analysis as more sensitive routine parameters for women.

The current knowledge about women in CVD offers a conflict of a reduced risk profile but worsened outcome in female patients. Women, especially in the pre-menopause state, have strong cards on their hands, but lose the potential at higher ages and during follow-up after an acute event. Thus, what are the main drivers behind this interaction in the multiplicity of hitherto revealed aspects? Along with factors associated with patients and health care systems, society-associated reasons might also play a role. Women have more caring responsibilities which hinders assess to rehabilitation and disease management programs. Is there a need for gender-specific prevention campaigns and risk stratification or follow-up programs? With the goal of an optimized individualized medicine, there is still a lot of space for gender equality in CV medicine.

## Figures and Tables

**Figure 1:**
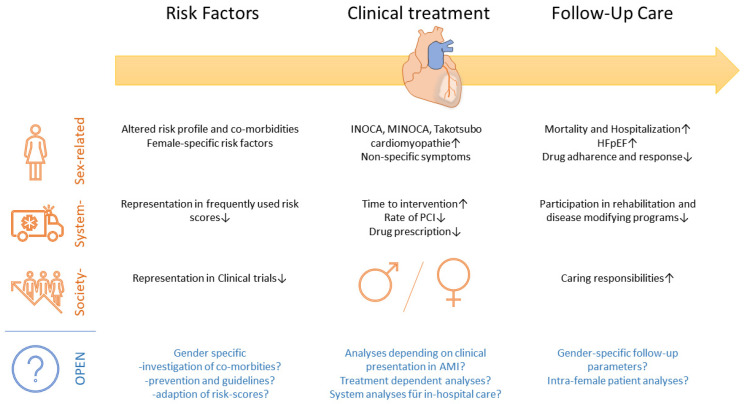
Adapted summarized aspects for gender-specific differences in cardiovascular disease [1], depending on risk factors, clinical treatment, and follow-up care complemented by relevant open questions and aspects.

## Data Availability

Not applicable.

## Abbreviations

AMI = acute myocardial infarction; CVD = cardiovascular disease, MACCE = major adverse cerebral- and cardiovascular events; PCI = percutaneous coronary intervention.
